# Alterations in cellular metabolisms after TKI therapy for Philadelphia chromosome-positive leukemia in children: A review

**DOI:** 10.3389/fonc.2022.1072806

**Published:** 2022-12-06

**Authors:** Chunmou Li, Luping Wen, Junchao Dong, Lindi Li, Junbin Huang, Jing Yang, Tianqi Liang, Tianwen Li, Zhigang Xia, Chun Chen

**Affiliations:** ^1^ Department of Pediatrics, the Seventh Affiliated Hospital of Sun Yat-Sen University, Shenzhen, Guangdong, China; ^2^ Department of Pharmacy, The Seventh Affiliated Hospital of Sun Yat-Sen University, Shenzhen, Guangdong, China; ^3^ Key Laboratory of Tropical Disease Control, Ministry of Education, Sun Yat-sen University, Shenzhen, Guangdong, China

**Keywords:** cellular metabolisms, TKI therapy, Philadelphia chromosome-positive, leukemia, children

## Abstract

Incidence rates of chronic myeloid leukemia (CML) and Philadelphia chromosome-positive (Ph+) acute lymphoblastic leukemia (ALL) are lower but more aggressive in children than in adults due to different biological and host factors. After the clinical application of tyrosine kinase inhibitor (TKI) blocking BCR/ABL kinase activity, the prognosis of children with CML and Ph+ ALL has improved dramatically. Yet, off-target effects and drug tolerance will occur during the TKI treatments, contributing to treatment failure. In addition, compared to adults, children may need a longer course of TKIs therapy, causing detrimental effects on growth and development. In recent years, accumulating evidence indicates that drug resistance and side effects during TKI treatment may result from the cellular metabolism alterations. In this review, we provide a detailed summary of the current knowledge on alterations in metabolic pathways including glucose metabolism, lipid metabolism, amino acid metabolism, and other metabolic processes. In order to obtain better TKI treatment outcomes and avoid side effects, it is essential to understand how the TKIs affect cellular metabolism. Hence, we also discuss the relevance of cellular metabolism in TKIs therapy to provide ideas for better use of TKIs in clinical practice.

## Introduction

Protein kinases (PKs), a class of enzymes, are able to transfer phosphate groups from ATP to the hydroxyl side chain of certain amino acid residues (1). PKs can be classified into tyrosine kinases (TKs) and serine/threonine kinases (STKs) based on the origin of the phosphorylated hydroxyl groups (2). TKs are essential cellular signaling enzymes regulating signal transduction pathways for metabolism, transcription, differentiation, proliferation, development, migration and apoptosis (3). TKs may be divided into two major classes: transmembrane receptors linked receptor tyrosine kinases (RTKs), like the PDGF receptors, and non-receptor tyrosine kinases (NRTKs), like c-SRC and BCR-ABL (4). Oncogenic mutations or overexpression of TK are a hallmark of cell cycle dysregulation often related to tumorigenesis (5) in hematological malignancies (6, 7), breast cancer (8), and non-small-cell lung cancer (9). Hence, TK inhibition (TKI) is regarded as a targeted treatment for cancer as it can selectively inhibit TK proteins and halt the proliferation and growth of tumor cells (3). At present, a variety of structurally different TKIs acting at singular or multiple targets like BCR-ABL, EGFR, VEGFR, PDGFR, KIT, and ALK, have been developed with minimal toxicities and good pharmacokinetics (10, 11).

A myeloproliferative tumor known as chronic myelogenous leukemia (CML) is characterized by unchecked proliferation of bone marrow myeloid progenitor cells resulted from the translocation of t (9:22) producing the hallmark BCR-ABL1, a constitutively active tyrosine kinase (12). Pediatric CML accounts for about 9% of leukemia in teenagers between the ages of 15 and 19 and around 2% of leukemia in children under the age of 15 (13, 14). However, previous studies showed that owing to the underlying biology and host characteristics, clinical presentations in children are often more aggressive than those in adults (14, 15). Similar to adult, the natural history of pediatric CML also progresses through three phases (16). The first and most prevalent stage is the chronic phase (CP), which is characterized by the absence of any subjective symptoms 3–5 years after diagnosis. The second stage is the accelerated phase (AP), during which aberrant granulocyte differentiation increases. The last stage is known as the blast crisis (BC), which is characterized by an expansion of undifferentiated blasts. Fortunately, TKIs can be used to treat patients in the CP effectively, leading to improved survival (17); however, the majority of AP and BC patients show no response to TKIs (18) because their growth is no longer influenced by BCR-ABL. Before the introduction of TKIs, pediatric CML was mainly treated with hematopoietic stem-cell transplantation (HSCT) and had an overall survival (OS) of about 64% at a median follow-up period of 6 years (19). In May 2001, the US Food and Drug Administration (FDA) approved Imatinib (IM, a small molecule TKI) for adult CML (20) and this strategy was successful in improving disease prognosis. In 2003, the US FDA authorized IM for pediatric (under 18 years of age) CML based on its effectiveness in adults (21). Since then, the OS of all children with CML treated with TKIs has improved to about 90% at the median 3-year follow-up period (19).

Philadelphia chromosome-positive acute lymphoblastic leukemia (Ph+ ALL) which is characterized by the t(9;22)(q34;q11) translocation and BCR-ABL1 fusion gene accounts for 3% to 5% of children with ALL (22, 23). Children with Ph+ ALL also have a more aggressive clinical presentation because of the secondary cytogenetic abnormalities and cooperative mutations such as IKZF1 deletions (22, 24). Ph + ALL is an adverse subtype of ALL with poor prognosis. Historically, less than half of children with Ph+ ALL survived when treated with chemotherapy with or without HSCT (25–27), while survival for children with ALL exceeds 90% in the same period (28). Fortunately, due to the success of TKI treatment in CML, TKIs was introduced for Ph+ ALL treatment, leading to an improved OS and event-free survival (EFS) rates in pediatric Ph+ ALL when combined with intensive chemotherapy (29, 30).

Despite the recent advancement of TKI therapies, drug resistance remains a problem in clinical anticancer treatment. In recent years, mechanisms of acquired resistance have been identified. TKI resistance in the treatment of CML can result from both BCR-ABL dependent and independent pathways (31). The majority of the BCR-ABL-dependent resistance (32–36) is mediated by the T315I “gatekeeper” mutation, BCR-ABL overexpression, MDR1 upregulation, and ABL kinase domain mutation, respectively. Currently, it is not so clear about BCR-ABL-independent resistance. Recent findings indicate that such TKI resistance may be influenced by the insensitivity of leukemia stem cells (LSCs) (37) and abnormal activation of the PI3K (38) and RAS/MAPK (39, 40) signaling pathways. To overcome IM resistance, second-generation TKIs, such as dasatinib (DAS) and nilotinib (NIL), were developed and they have activity against most IM-resistant BCR-ABL1 mutants (41). But, they are not able to overcome ABL-T315I-induced resistance. Then, ponatinib, the third-generation TKI, has been developed and is effective against T315I-mutated Ph+ leukemias (42), but the risk of life-threatening cardiovascular side effects limits its clinical application (43). On the other hand, due to the need of growth and development, the long-term clinical safety of TKIs has to be considered. In recent years, several long-term side effects have been reported, including growth deceleration (44), dysregulation of bone (45, 46), and decreased fertility (47, 48).

To achieve better treatment outcomes while avoiding drug resistance and side effects, a deeper comprehension of TKIs’ mechanism is necessary. Therefore, this review discusses the metabolic pathways alterations after TKIs therapy in children, including the following five aspects: glucose metabolism, lipid metabolism, amino acid metabolism, nucleotide metabolism, and immunometabolism.

## Glucose metabolism

Glucose metabolism, which serves as a significant source of energy for cell development, includes glycolysis pathway, pentose phosphate pathway (PPP), oxidative phosphorylation and serine synthesis pathway (SSP) (49). It is known that in aerobic settings, intracytoplasmic glycolysis provides energy first, followed by mitochondrial oxidative phosphorylation. When oxygen is scarce, cells depend on glycolysis instead of the oxygen-consuming TCA cycle to produce energy (49). However, Otto Warburg found that even in the presence of enough oxygen, cancer cells prefer to engage in aerobic glycolysis, generally referred to as “Warburg effect” or “aerobic glycolysis”, to produce ATP and metabolic intermediates (50). Studies showed that although glycolysis produces ATP per glucose molecule considerably less effectively than oxidative phosphorylation, the production rate increases significantly (51). Besides, this reprogramming of glucose metabolism provides additional macromolecular precursors such as acetyl-CoA, glycolytic intermediates and ribose which meet the needs of fast growth and proliferation of cancer cells (52). Like other malignancies, the aberrant cellular metabolism also occurs in CML cells. The BCR-ABL oncoprotein can boost glucose uptake and glycolysis and overexpress glucose transporter-1 (GLUT-1) to influence metabolism (53). Furthermore, the PI3K/Akt/mTOR pathway is considered to be responsible (54). Since the introduction of the first BCR-ABL TKI, IM (Gleevec, previously STI571), the effects of BCR-ABL TKIs on glucose metabolism in tumor cells have been explored.

Using [1,2-13C2] glucose as the single tracer with biological mass spectrometry, J Boren et al. demonstrated that (55) by lowering hexokinase and glucose-6-phosphate 1-dehydrogenase activity and changing pathway carbon flux of the pentose cycle in K562 human myeloid blast cells, IM reduced the use of glucose carbons for *de novo* nucleic acid and fatty acid synthesis. In 2004, Gottschalk S et al. used magnetic resonance spectroscopy to examine changes in endogenous metabolites, energy status, and glucose metabolism of human BCR-ABL+ cells (CML-T1 and K562) and BCR-ABL- cells (HC-1) following IM therapy (56). They found that the “Warburg effect” was reversed in BCR-ABL+ cells at the appropriate therapeutic doses of IM (0.1-1.0 μmol/L) by switching glucose metabolism from anaerobic glycolysis to the mitochondrial Krebs cycle. In this situation, BCR-ABL+ cells decreased the glucose uptake from the media but the glucose metabolism in mitochondrial increased, leading to elevated absolute concentrations of the high energy phosphate nucleoside triphosphate (NTP). Subsequently, Barnes K et al. investigated the function of BCR-ABL-induced glucose transport regulation anomalies in CML (9). This work showed that by upregulating the GLUT-1 glucose transporter’s expression on the cell surface, BCR-ABL-expressing cells may accelerate the absorption of hexose. Interestingly, IM treatment leads to a 90% internalization of the cell-surface GLUT-1 transporters, substantially decreasing hexose uptake in BCR-ABL-expressing cells (57). These findings suggested that reversing the aerobic glycolysis and inhibiting glucose transport significantly contributes to IM’s anti-tumor effects.

Pyruvate-Kinase (PK), as an enzyme, catalyzes the last stage of glycolysis, converting phosphoenolpyruvate and ADP into pyruvate and ATP (58). According to previous literature, the Warburg effect is accomplished by regulating expression of the embryonic M2 isozyme of PK (PKM2), rather than the M1 isozyme (PKM1) expressed in normal cells, through an alternative splicing repressor polypyrimidine tract-binding protein1 (PTBP1) (59, 60). A study suggested that IM inhibits glycolysis through the inhibition of phosphorylation of BCR-ABL and the down-regulation of miR-124/PTBP1/PKM2 signaling (61). Through downregulation of PTBP1, IM changes PK isoforms from PKM2 to PKM1, leading to reversal of the Warburg effect (61). Apart from this, Damaraju VL et al. reported how TKIs reduce glucose uptake by evaluating the interaction of TKIs with GLUT-1 in the human nasopharyngeal carcinoma cell line (FaDu) and GIST-1 cells. They discovered that [3H]2-deoxy-d-glucose ([3H]2-DG) and [3H]Fluoro-2-deoxy-D-glucose (FDG) uptake were competitively suppressed by IM and NIL, and that IM had reversible [3H] FDG uptake inhibition whereas NIL did not (62). Additionally, molecular modeling demonstrated that TKIs impair GLUT-1’s ability to take up glucose by interacting with the glucose binding site *via* hydrogen bonds and van der Waals interactions (62).

Intrinsic metabolic differences between IM sensitive and resistant cell lines were also previously characterized. A study showed that IM treatment in sensitive BCR-ABL positive cells (K562-S, LAMA84-S) leaded to the reduction of glucose absorption and lactate generation and the enhancement of oxidative TCA cycling (53). On the other hand, the drug-induced IM resistant cells (K562-r and LAMA84-r) displayed a highly glycolytic metabolism with increased glucose absorption and lactate generation. Additionally, in IM-resistant cells, oxidative synthesis of RNA ribose from 13 C-glucose using glucose-6-phosphate dehydrogenase was decreased, while the non-oxidative transketolase pathway was boosted (53). In line with the literature mentioned above (57), in IM-treated sensitive cells, GLUT-1 moved from the plasma membrane to the intracellular fraction leading to reduced glucose uptake, while GLUT-1 remained at the plasma membrane in IM-resistant cells (53). However, different from the finding in the drug-induced IM resistant cells, Ko BW et al. reported that the KBM5-T315I cells which acquired drug resistance resulting from the T315I mutation have metabolically suppressive status compared to KBM5 cells (IM-sensitive) (63). KBM5-T315I cells showed low glycolytic activity, decreased fatty acid synthesis and reactive oxygen species (ROS) generation potentially participating to the reduced proliferative activity of KBM5-T315I cells. The researchers came to the conclusion that the decreased expression of glycolysis-related genes and ROS levels might be responsible for reduced growth ability of KBM5-T315I CML (63). These biological and metabolic characteristics of CML cells with different resistance mechanisms should be take into account in future studies overcoming the IM resistance.

As indicated previously, glycolysis was extremely important for B-ALL cells. T Liu et al. reported that 2-deoxyglucose (2-DG) suppressed aerobic glycolysis, leading to the inhabitation of B-ALL cell growth, the increasing of the pro-apoptotic protein Bim and re-sensitization of B-ALL cells to the tyrosine kinase inhibitor DAS *in vivo (* 64). Apart from this, deletion of GLUT-1 partially inhibits glucose uptake (64). Through metabolic reprogramming, the decreased glucose transport capacity was sufficient to decrease anabolism and promote catabolism in B-ALL cells. As a result, GLUT1-deficient B-ALL cells were unable to accumulate *in vivo*, and GLUT-1 depletion inhibited leukemia progression. These data suggested that inhibition of aerobic glycolysis and glucose uptake by GLUT-1 could be plausible adjuvant approaches for B-ALL therapies. In another study, researchers found that TKI treatment creates a new metabolic state in leukemic cells that is highly sensitive to specific mitochondrial perturbations. As a result, patients with BCR-ABL+ leukemia may respond better to TKI when receiving adjuvant therapy with targeting mitochondrial metabolism (65). Since TKI treatment changed glucose metabolism in BCR -ABL+ cells from anaerobic glycolysis to the mitochondrial tricarboxylic acid cycle, they indicated that oligomycin A, a mitochondrial ATP synthase inhibitor, greatly promotes TKI sensitivity in leukemia cells at very low concentrations *in vitro*. In a mouse model, oligomycin A enhanced the ability of TKI to eliminate BCR-ABL+ leukemia cells (65). In spite of strong suppression of glycolysis, Shinohara H et al. found that by upregulating carnitine O-palmitoyltransferase 1 (CPT1C), the rate-limiting fatty-acid oxidation (FAO) enzyme, IM triggers compensatory FAO, which enabled glucose-independent cell viability. AIC-47 suppresses CPT1C expression and directly inhibits the metabolism of fatty acids and. Combined with AIC-47, IM enhanced the attack on cancer energy metabolism, leading to an increased cytotoxicity (61). Overall, these studies illustrate that metabolic reprogramming after TKIs treatment could be a potential therapeutic target.

In addition to BCR-ABL cells, the effects of BCR-ABL TKIs on other cells were also investigated. Recent studies reported the influences of IM and DAS on skeletal muscle cell metabolism (66, 67). Damaraju VL et al. showed 2-deoxy-D-glucose absorption was suppressed by to almost 50% in C2C12 murine skeletal muscle cells pre-incubated for 15 min with IM. Moreover, in a skeletal muscle cell model, IM lowered energy generation and mitochondrial function by inhibiting mitochondrial complex V activity and nucleoside absorption. This may contribute to tiredness, one of the most prevalent side effects of TKIs (66). In addition to inhibiting glucose transport proteins and metabolic enzymes, IM also exerts antidiabetic effects by protecting against β-cell death and ultimately increasing insulin production in a mouse model (68). In CML patients, Fitter S. et al. found a 3-fold increase in plasma adiponectin concentrations after three months of IM treatment; adiponectin elevation enhanced glucolipid metabolism, which explains why diabetes improved after IM treatment (69).

## Lipid metabolism

There are two mechanisms by which mammals acquire lipids: *de novo* synthesis and direct exogenous uptake. The *de novo* lipogenesis pathway is restricted to hepatocytes and adipocytes in normal tissue; however, cancer cells can also reactivate this pathway even with exogenous lipids (70). An elevated of lipid uptake, storage and lipogenesis was reported in a variety of cancers, contributing to rapid tumor growth (71, 72). Research suggested that lipid metabolism in cancer cells is regulated by PI3K/Akt/mTOR pathway (24). Sterol regulatory element-binding protein 1c (SREBP-1c), a transcription factor that promotes lipid synthesis *de novo*, is controlled by mTORC1 (70, 73). In spite of lipids being widely used as cancer biomarkers, little is known about TKIs’ impact on lipid metabolism and pathways.

Previous studies have suggested that exposure to the first-generation TKI (IM) may lead to a reduction in cholesterol and triglycerides in people and animal models, as well as a better serum lipid profile, while the second-generation TKIs may cause a worse metabolic profile (74–77). A cohort study researched how first- and second-generation TKIs affected the patients with CML’s glucose and lipid metabolism. They discovered that compared to the IM and DAS groups, the NIL group had substantially higher fasting plasma glucose, insulin, C-peptide, insulin resistance, total cholesterol, and low-density lipoprotein (LDL) cholesterol levels (76). In a translational mouse model, plasma cholesterol and atherosclerosis areas were reduced by IM and ponatinib, while they were not affected by NIL. On the other hand, IM showed a beneficial cardiovascular risk profile compared to NIL and ponatinib (74).

It is not entirely clear how IM reduces lipid levels. The PDGF receptor (PDGFR) inhibitory action of IM has been proposed as a potential reason. The phosphorylation of LDL receptor-related protein (76) may be facilitated by excessive PDGFR expression, leading to atherosclerosis brought on by cholesterol. However, NIL, which also inhibits PDGFR, was found to significantly increase lipid levels in patients, rendering this explanation unsatisfactory (76). Ellis M et al. explored the possible biological mechanisms behind the lipid-lowering effects of IM in CML. Results indicated that two genes, apobec1 that inhibit lipid synthesis and LDL-R that promote clearance of circulating LDL, are significantly induced by IM. In addition, IM induced HMG-coAR expression, which regulates hepatic cholesterol synthesis (78). To elucidate the effects and mechanisms of TKIs on lipid metabolism, additional studies will be needed.

Studies on BCR-ABL TKIs and lipid metabolism are limited so far. The aforementioned study by Gottschalk et al. reported that phosphocholine concentrations, which are known to be raised in all rapidly proliferating malignant cells, were significantly reduced in IM-treated BCR-ABL+ cells (56). Similarly, subsequent study, which assessed a global metabolic profile including lipid metabolism of human leukemia cell after incubation with IM, showed that IM-treated K562 cells had a decreased concentrations of phosphocholine (PC) and phosphatidylcholine (PtdCho) (79). Following the first week of IM treatment, this reduction was significant and even increased after 2 -4 weeks. Polyunsaturated fatty acids (PUFAs) signal intensity increased after 2 and 4 weeks of treatment with increasing apoptosis rate. Furthermore, the amount of methylene/methyl (CH2/CH3) resonances of fatty-acid chains also enhanced (31). Previous studies have reported the decrease in PtdCho concentration with advancing apoptosis stages (80). Additionally, the accumulation of PUFAs as well as increased CH2 and CH3 resonances of free fatty acids is two other characteristics associated with cell death (81). Collectively, these lipid metabolic events following TKI treatment in BCR-ABL+ cells showed pathways linked to a continuous process of cell death.

## Amino acid metabolism

Amino acids are basic units for protein synthesis in the organism and can divide into two groups: essential amino acids and non-essential ones. Essential amino acids cannot be synthesized by humans and can only be provided by food sources (82), including phenylalanine, valine, threonine, tryptophan, methionine, leucine, isoleucine, lysine and histidine (can be synthesized in adults). Non-essential amino acids include arginine, cysteine, glycine, glutamine, proline, tyrosine, alanine, aspartic acid, asparagine, glutamate and serine which can be synthesized in the body. After malignant transformation, tumor cells have an increased demand for amino acids, which can be utilized as intermediates in many metabolic pathways (83). According to studies in recent years, under genotoxic, oxidative, and nutritional stress, amino acids can act as metabolic regulators to promote cancer cell proliferation and survival (84, 85). Among them, studies on glutamine, serine, glycine and branched-chain amino acids (BCAAs) have drawn more attention.

## Glutamine

As mentioned above, TKIs can efficiently hamper glucose metabolism in Ph+ leukemia. But, due to compensatory metabolic pathway activation, glycolysis inhibition alone frequently falls short of eliminating cells (86, 87). Glutamine is the most prevalent amino acid in human plasma and the second only to glucose in the metabolism of tumor cells (88). This metabolic alteration is frequently observed in cancer (89). Glutamine may provide its nitrogen and carbon to a variety of mechanisms in cancer cells, including energy production, macromolecular synthesis, and signal transmission (90).

Through the transporters (e.g., SLC1A5 or ASCT2), glutamine is brought into the cytoplasm where it is converted to glutamate by the enzyme glutaminase(GLS) (91). An earlier work shown that human B cell Burkitt lymphoma cell line P493 cells may use glutamine to carry out glucose-independent mitochondrial oxidative phosphorylation in the presence of low oxygen levels (92). Combined transcriptome and metabolome profiling, Pallavi Sontakke et al. found that (93), despite the prominent glycolysis, BCR-ABL positive cells also undertake glutaminolysis, which was demonstrated by elevated intracellular glutamine levels both in normoxia and hypoxia. In agreement with these findings, they also discovered that both protein and RNA levels of the glutamine importer SLC1A5 increased in BCR-ABL-expressing cells. Given this circumstance, glutamine may play a significant function as an additional source of carbon in the replenishment of tricarboxylic acid cycle (TCA) metabolite. Other researches have demonstrated that this is indeed the case. Anne Trinh et al. found that, after the treatment of IM, survived CML cells can continue to consume glutamine to create alphaKetoGlutarate, a TCA intermediate, that keeps the state of high mitochondrial oxidative metabolism (94). After that, they found that the combination of Kidrolase (an FDA-approved drug of L-asparaginase) and IM can deplete extracellular glutamine and therefore restrict mitochondrial metabolism. Finally, they discovered that this combination stimulates the intrinsic apoptotic pathway, effectively killing CML cells. Furthermore, TKIs are unable to eradicate the leukemia stem cells (LSCs) and/or progenitor cells that could cause relapses (95). In order to kill LSCs, Anne Trinh et al. also provided evidence that both glycolysis and glutamine-dependent mitochondrial metabolism required to be impaired (94). To prevent recurrence and achieve a longer OS of children, it may be an interesting therapeutic approach to eradicate LSCs by combining TKI and mitochondrial inhibitors.

## Serine and glycine metabolism

Serine is well known as the one-carbon source in the methionine cycle and folate cycle and contributes to nucleotide synthesis, methylation reactions, and production of NADPH, an antioxidant defense mechanism (96, 97). Serine and glycine can be imported from the extracellular environment produced by the *de novo* serine synthesis pathway (SSP) (97). The SSP, starting from the glycolytic metabolite 3-phosphoglycerate (3-PG), is composed of three steps. First, phosphoglycerate dehydrogenase (PHGDH) converts 3-PG into 3-phosphohydroxypyruvate; then, phosphoserine-amino transferase (PSAT-1) converts 3-phosphohydroxypyruvate into phosphoserine; last, phosphoserine phosphatase (PSPH) eventually catalyzing the dephosphorylation of phosphoserine into serine (98). As mentioned above, as a key metabolite to support cell proliferation, increasing serine supply is required to sustain cancer progression (99). In various cancers, to meet the large serine demand for survival, the three SSP enzymes (PHGDH, PSAT, and PSPH) are all highly expressed (100). In addition, recent work on cancer metabolomics has shown that unexpected increased reliance on glycine metabolism was discovered in rapid proliferation cancer cells and this phenotype that was not observed in rapidly proliferating nontransformed cells (101). Interestingly, an animal experiment found that restriction of serine and glycine intake can inhibit tumor growth and extend the survival time of tumor-bearing mice (102). Hilal Taymaz-Nikerel et al. reported some new multi-omics findings in yeast on the mechanism of IM, using the model organism Saccharomyces cerevisiae (103). They performed the whole-genome analysis of the transcriptional response of yeast cells *via* flux-balance analysis (FBA) and modular analysis of protein/protein interaction network which consist of proteins encoded by differentially expressed genes (DEGs). FBA indicated that IM alters multiple metabolic pathways by decreasing and increasing the fluxes of reactions and the fluxes related to metabolic pathway of glycine and serine were increased. However, through proteomics and metabolomics profiling of IM-resistant CML cells (ImaR), a previous study showed that serine-glycine-one-carbon metabolism and proline synthesis were enhanced in KU812 ImaR cells (104). In summary, IM can indeed suppress the proliferation of CML cells and induce the increasing of metabolic pathway of glycine and serine in parallel. Based on these, could we then assume that over-represented glycine and serine might counteract the inhibitory effect of IM and promote CML cell survival and chemoresistance? If so, suppressing the metabolism of glycine and serine could possibly serve as a novel therapeutic target.

## BCAAs

For mammals, the BCAAs are necessary amino acids, including leucine, isoleucine, and valine. In mammalian proteins, about 63% of the hydrophobic amino acids are BCAAs (105) and may only be attained from food intake and recycled scavenged protein (106). After being catabolized by enzymes, intracellular BCAAs can provide nitrogen and carbon groups to take part in the synthesis of biomass, energy production, nutritional signaling, and epigenetic regulation (107). In humans, branched-chain amino transferases (BCATs) comprise two compartment-specific BCAA transaminases (BCAT1 and BCAT2) and can produce glutamate and the corresponding branched-chain α-ketoacids (BCKAs) (108, 109). When the transamination and transfer of nitrogen to α-ketoglutarate (α-KG) by BCATs initiate, catabolism of BCAAs begins. BCAAs and BCKAs often coexist in a balanced state, however this is not the case with malignancies. By fluorescent markers unique to each amino acid, Hattori A et al. measured the amino acid contents in CML-initiating cells isolated from a BC-CML mouse model. They discovered that BCAA levels were significantly higher in these cells (110). They later found that increased BCAT1 (or cBCAT), which may catalyze the conversion of a BCKA plus glutamic acid (Glu) into a BCAA and a -KG, may be a factor in this heightened BCAA metabolism. Additionally, they discovered that BC-CML-initiating cells had higher levels of BCAT1 mRNA, and that transduction of shRNA targeting BCAT1 mRNA reduced intracellular BCAA levels, which impeded the ability to form colonies *in vitro*. Through multi-omic investigations in yeast, Hilal Taymaz-Nikerel et al. found that (103) after IM treatment, the fluxes of the processes involved in the production of various amino acids, such as isoleucine, lysine, histidine, threonine, and valine, were drastically downregulated. In addition, Miriam G. Contreras Mostazo et al. indicated that KU812 ImaR cells might consume more BCAAs than parental cells in normoxia (104). According to these findings, inhibiting BCAA metabolism may be a promising therapeutic strategy for reducing ImaR cells.

## Others

Methionine and homocysteine, two sulfur-containing amino acids, are the primary precursors of glutathione, a tripeptide that lowers reactive oxygen species (ROS) and upholds redox equilibrium (111). Additionally, Hilal Taymaz-Nikerel et al. discovered that (103) following IM therapy, the reaction fluxes through the production of methionine and cysteine, as well as the absorption of sulfate, were determined to be drastically decreased.

## Nucleotide metabolism

In all areas of life, nucleotide metabolism is a critical activity. In order to enable cell proliferation (112), nucleotides, a type of biological information macromolecule, are primarily used as the raw materials for the synthesis of nucleic acids. Nucleotides comprise of both purine (adenine and guanine) and pyrimidine (cytidine, uridine and thymidine). Therefore, inhibitors of purine or pyrimidine synthesis have also been applied in hematological malignancies (113).

**Figure 1:**
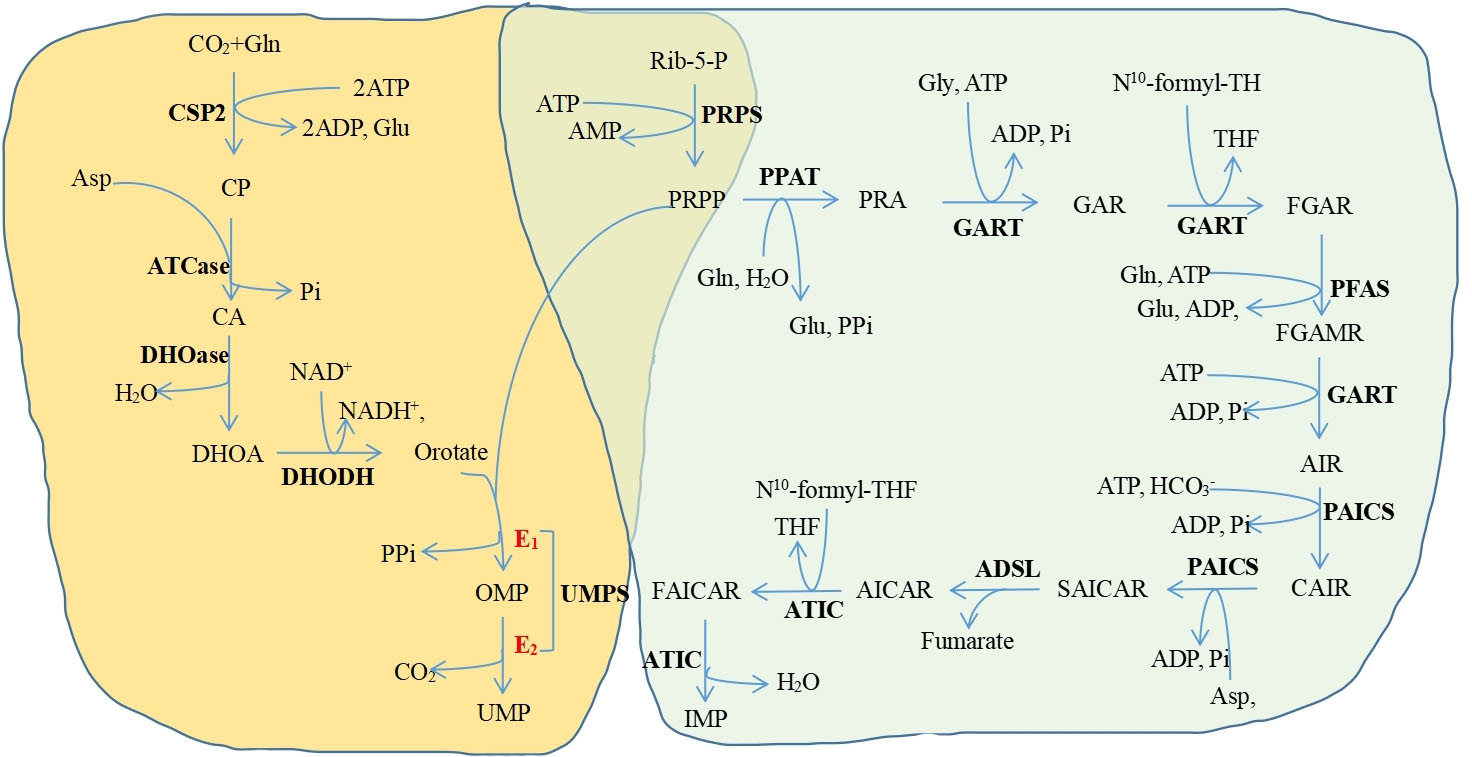
The de novo pyrimidine and purine synthesis pathways. Yellow background, the de novo pyrimidine synthesis pathway; green background, the de novo purine synthesis pathway Purines and pyrimidines get synthesized separately but they have one same thing. PRPP Gl. Glutamine, CSP2 Carbamoyl-phosphate synthetase 2: Glu, Glutamate: CP. Carbamoyl phosphate; ATCase, Aspartate transcarbamylase; CA, N-carbamoyl-L-aspartate DHOase. Dihydroorotase. DHOA. Ddihydroorotate, DHODH. Dihydroorotate dehydrogenase; OMP Orotidine 5'-monophosphate: UMPS. Undine monophosphate synthetase: UMP. Uridine monophosphate: Rib-5-p. Ribose-5-phosphate: PRPS. Phosphoribosylpyrophosphate synthetase; PRPP 5-phosphonbosyl-1-pyrophosphate: PPAT. Phosphoribosylpyrophosphate amidotransferase; PRA, Phosphoribosylamine, Gly. Glysine, GART Glycinamide ribonucleotide formyltransferase; GAR, Glycinamide ribonucleotide, THF. Tetrahydrofolate; FGAR, N-fotuvlelycinamide boucleotide, PEAS, Phosphoribosylformylglycinamidine synthase; FGAMR, N-formylglycinamidme ribonucleotide, AIR, Annnoimidazole ribonucleotide: PAICS, Phosphoribosylaminoimidazole carboxylase, phosphoribosylaminoimidazole succinocarboxamide synthetase: CAIR. Carboxyaminoimidazole nbonucleotide; Asp. Aspartate: SAICAR, Nsuccinocarboxamide-5-aminoimidazole ribonucleotide, ADSL. Adenylosuccinate lyase: AICAR 5-aminoimidazole-4-carboxamide ribonuckotide ATIC 5-aminoimidazole-4-carboxamide rbouucleotide transfonuylase FAICAR 5-formamido-4-imidazolecarboxamide ribonucleotide, IMP. Inosine-5-monophosphate: PP. Pyrophosphate: ATP. Adenosine-S-triphosphate: ADP Adenosine 5'-diphosphate; AMP Adenosine 5'-monophosphate; Pi. Phosphate, ${\text{HCO}}_{3}^{-}$ . Hydrogen carbonic acid, CO., Carbon dioxide.

Purines and pyrimidines get synthesized separately but they have one same thing: 5-phosphoribose-1-pyrophosphate (PRPP), which is a donor of phosphate and ribose sugar and is an active form of ribose generated from ribose 5-phosphate (**Figure 1**). In purine biosynthesis, the primary source of nucleotides *in vivo* is *de novo* synthesis, and negative feedback mostly controls the rate of nucleotide synthesis (114). In the process of making purines, PRPP is transformed into inosine monophosphate (IMP), which needs 6 ATP, glutamine, glycine, and aspartate. Then, IMP can be converted into guanosine monophosphate (GMP) or adenosine monophosphate (AMP) through different enzymes. The purine nucleotide salvage mechanism is easier and uses less energy than the *de novo* synthesis method (115, 116). However, since the lack of the enzyme system which can synthesize purine nucleotides from scratch, the brain and bone marrow can only conduct the salvage approach to generate purines (117, 118). In addition, previous studies have shown that the *de novo* nucleotide synthesis pathway is also usually found in proliferating cells, including immune cells and cancer cells (119, 120). An earlier study (121) revealed that a higher level of *de novo* purine synthesis was identified in the leukocytes and plasma of newly diagnosed CML patient due to the enhancement of 5-aminoimidazole-4-carboxilic acid ribonucleoside (CAIR). They discovered that the majority of the purine levels had stabilized toward the control values after IM and NIL therapy. However, adenosine 5’-monophosphate, guanine, guanosine, guanosine 5’-monophosphate, and inosine 5’-monophosphate did not change toward the control values in the patients receiving DAS.

Pyrimidine synthesis is also split into *de novo* synthesis and salvage pathways, just like purine biosynthesis (122). In addition to PRPP, pyrimidine biosynthesis also needs aspartate, glutamine, bicarbonate, and 2 ATP (123). Leukocytes from newly diagnosed patients had elevated levels of pyrimidine metabolism, particularly cytosine, cytidine 5’-monophosphate, cytidine 2’,3’-cyclic phosphate, and uridine 5’-monophosphate (121). Most of these metabolites returned to control levels after TKI therapy. But, some pyrimidine metabolites (cytidine, cytidine 5’-triphosphate, and uridine) were still present at similar levels in the patients receiving DAS.

By multi-omics, Taymaz-Nikerel et al. revealed that, after IM treatment, the fluxes of the processes involved in the production of purine and pyrimidine nucleotides were dramatically downregulated in yeast (103).

## Immunometabolism

Historically, abnormal energy metabolism has been known as a hallmark of cancer (124). Recent research has revealed that immune cells undergo metabolic reprogramming during the activation and differentiation processes, giving rise to the notion of “immunometabolism” (125). The field of immunometabolism is about how metabolic processes affect immune cell functions in physiological and pathological situations (126). In cancer cells, complex and dynamic metabolic reprogramming can make themselves to accommodate tumor microenvironment (TME), which can restrict the biosynthetic and bioenergetic demands for growth (127). Additionally, cancer cell metabolism not only aggressively competes for essential resources but also produces metabolic byproducts that affect immune cell activation, fitness, and effector function in a direct or indirect ways (128–131). Instead of inhibiting or killing cancer cells, these defective immune cells even may become tumor-supporting cells to speed up the spread and invasion of cancer (125).

In recent years, the way to manage tumor patients has significantly changed as a result of tumor immunotherapy (132). During the treatment, TKIs do not only target BCR-ABL1 but also inhibit additional targets such c-KIT, TEC, SRC, FLT3, Lck, and mitogen-activated kinases (MAPK) (133). This “off-target” effect can alter immune responses, both harmful and beneficial. Lisa Christiansson et al. found that (134) IM and DAS can both lower immune escape mechanisms by reducing the number of myeloid suppressor cells and the inhibitory factors arginase 1 (Arg1), Myeloperoxidase (MPO), and IL-10. According to researches, the proportion of Myeloid-derived suppressor cells (MDSC) and the blood concentrations of Arg1 and inducible nitric oxide synthase (iNOS) were both considerably higher in CML patients at diagnosis and significantly lower after TKI therapy (135). ZIYUAN LU et al. found that (136) following treatment with a TKI (IM, DAS or NIL), Total T cells, Tregs (whose decline became more pronounced over time), CD4+ T cells, and CD8+ T cells all reduced to varying degrees in CML patients. They also revealed that IM and DAS may be more effective than NIL on decreasing the number and function of Tregs. Silke Appel et al. (137) reveals that IM inhibits dendritic cell (DC) differentiation and function *via* Akt and nuclear factor-κB signal transduction. After then, Daniela Dorfel et al. (138) discovered that both IM and NIL considerably and similarly hampered monocyte differentiation into DCs, with only a partial recovery after TKI discontinuation.

Taking into account the long-term side effect and the cumulative cost of TKI therapy in children, it is clear that it is important to avoid lifelong treatment with TKIs. As mentioned above, LSCs have high correlations with tumor recurrence and it is crucial to eliminate LSCs. One potential solution is to stop after a certain period of deep molecular remission and restore normal immune functions, especially the NK cells (139–142). Previous studies indicated that patients with stable NK cell counts accompanied by higher cytotoxicity and increased killing capacity are more inclined to get sustained treatment-free survival (142, 143). Previous study showed that following TKI treatment, the proportion of effector NK cells were increased (135). The results indicate that the number and killing capacity of NK cells may be utilized to further assess the risk of TKIs discontinuation. Geoffrey D. Clapp et al. suggested that (144) carefully timed vaccines may stimulate the patient’s immune system to drive the residue LSCs to extinction. In recent trials of Ph+ ALL, Schultz KR et al. (145) used limited duration, more intensive chemotherapy in combination with TKIs for children and adolescents and had an initial observation of substantially good outcomes. It may be another option to eliminate the CML LSCs.

## TKI adverse effects in children

Due to off-target effects, IM can cause substantial growth abnormalities in children with CML (146–148) by a direct effect on the growth plate (149), acquired growth hormone deficiency and disturbing the GH : IGF-1 axis (150). Second- and third-generation TKIs in children have less clinical data, but DAS appears to have a similar impact on growth (151).

TKIs’ teratogenic potential makes them potentially harmful to pregnant women as well (152). Associated studies showed that (153) female partners of male patients are not at risk for pregnancy-related complications and TKI should be continued. For female patients, contraception should be planned during TKI, so when in major molecular remission for more than two years, pregnancy can be planned. In addition, NIL appears to be the safest (153).

As recommendations for monitoring and supportive care in children with CML receiving TKI therapy, height, weight, gonadotropins, sex steroids, thyroid function (TSH, free T4), echocardiogram and electrocardiogram be examined routinely (14).

## Summary

Overall, TKI is obviously regarded to be the most effective kinase inhibitor for CML treatment nowadays and the role of TKI in Ph+ ALL treatment has also attracted increased attention. Recently, an increasing number of studies have demonstrated that TKIs can affect the normal metabolism of Ph+ leukemia cells to achieve therapeutic purposes. But in children, there are still many difficulties to surmount, such as the off-target effects, drug tolerance, disease recurrence, adverse effects, cumulative cost. In this study, we perform a more detailed analysis about cellular metabolism alterations after TKI therapy, but further research is needed because so many interested targets can be combined with TKI therapy to provide great benefits to Ph+ leukemia children.

## Author contributions

JH, JY, TWL, LL, TQL, and ZX performed the collection and interpretations of all relevant literature. CL and LW write the manuscript. CC and JD critically read and revised the manuscript. All authors contributed to the article and approved the submitted version.
